# Biochars from olive mill waste have contrasting effects on plants, fungi and phytoparasitic nematodes

**DOI:** 10.1371/journal.pone.0198728

**Published:** 2018-06-07

**Authors:** Roberta Marra, Francesco Vinale, Gaspare Cesarano, Nadia Lombardi, Giada d’Errico, Antonio Crasto, Pierluigi Mazzei, Alessandro Piccolo, Guido Incerti, Sheridan L. Woo, Felice Scala, Giuliano Bonanomi

**Affiliations:** 1 Department of Agricultural Sciences, University of Naples Federico II, Portici, Naples, Italy; 2 Task Force on Microbiome Studies, University of Naples Federico II, Naples, Italy; 3 Institute for Sustainable Plant Protection, National Research Council, Portici, Naples, Italy; 4 Interdepartmental Research Centre on Nuclear Magnetic Resonance for the Environment, Agro-Food, and New Materials (CERMANU), University of Naples Federico II, Portici, Naples, Italy; 5 Department of Agri-Food, Environmental and Animal Sciences, University of Udine, Udine, Italy; 6 Department of Pharmacy, University of Naples Federico II, Naples, Italy; RMIT University, AUSTRALIA

## Abstract

Olive mill waste (OMW), a byproduct from the extraction of olive oil, causes serious environmental problems for its disposal, and extensive efforts have been made to find cost-effective solutions for its management. Biochars produced from OMW were applied as soil amendment and found in many cases to successfully increase plant productivity and suppress diseases. This work aims to characterize biochars obtained by pyrolysis of OMW at 300 °C to 1000 °C using ^13^C NMR spectroscopy, LC-ESI-Q-TOF-MS and SEM (Scanning Electron Microscopy). Chemical characterization revealed that biochar composition varied according to the increase of pyrolysis temperature (PT). Thermal treated materials showed a progressive reduction of alkyl C fractions coupled to the enrichment in aromatic C products. In addition, numerous compounds present in the organic feedstock (fatty acids, phenolic compounds, triterpene acids) reduced (PT = 300 °C) or completely disappeared (PT ≥ 500 °C) in biochars as compared to untreated OMW. PT also affected surface morphology of biochars by increasing porosity and heterogeneity of pore size. The effects of biochars extracts on the growth of different organisms (two plants, one nematode and four fungal species) were also evaluated. When tested on different living organisms, biochars and OMW showed opposite effects. The root growth of *Lepidium sativum* and *Brassica rapa*, as well as the survival of the nematode *Meloidogyne incognita*, were inhibited by the untreated material or biochar produced at 300 °C, but toxicity decreased at higher PTs. Conversely, growth of *Aspergillus*, *Fusarium*, *Rhizoctonia* and *Trichoderma* fungi was stimulated by organic feedstock, while being inhibited by thermally treated biochars. Our findings showed a pattern of association between specific biochar chemical traits and its biological effects that, once mechanistically explained and tested in field conditions, may lead to effective applications in agriculture.

## Introduction

Cultivation of olive and oil production are important agricultural activities in countries of the Mediterranean basin [1, 2], with Spain, Italy, Greece, Turkey and Tunisia representing the world biggest producers. Oil extraction from olives generates large amounts of waste by-products that can adversely affect soil microbes, water resources and plants when disposed in the environment [3]. The management of olive processing waste has received considerable attention in the last decades [4, 5]. The physicochemical properties of the residues depend on the method used for oil extraction. Since the early ‘90s, the modern two-phase technology has almost replaced the traditional pressing and the three-phase centrifugation system, thus reducing by 75% the olive mill wastes [3]. This system guaranteed less water consumption and generated a solid waste (also called “alperujo”, thereafter indicated as OMW), showing particular properties, such as high moisture and carbohydrate concentration [6]. Different methods have been proposed to valorize this solid OMW, including physicochemical treatments for second extraction of oil or energy recovery [6, 7], direct soil application as amendment [8], solid state fermentation for production of enzymes, livestock feed or fuel [9, 10], composting [11], or extraction of valuable compounds, such as pectins, polyphenols and fibers [12].

Pyrolysis is the process of anaerobic thermal decomposition at high temperatures that leads to the production of solid products (chars). “Biochars” are chars applied as a soil amendment to improve productivity, preserve carbon reserves or filter percolating soil water [13]. The original feedstock composition and thermal process conditions (i.e. temperature, heating rate, etc.) influence the characteristics of biochars [14, 15, 16, 17], thus determining the best option for its final use [18]. Chars with high calorific values (also known as charcoals) are successfully applied to produce energy, while those showing high porosity and abundance of aromatic compounds may be useful to decontaminate polluted soils and waters [14, 19, 20]. The benefits of biochar on crop performance have been also well documented [21]. These are related to different characteristics and abilities to: i) increase soil pH due to alkaline features; ii) improve the soil water regime due to improved water retention capacity; iii) detoxify many xenobiotic and allelopathic substances; iv) stimulate the growth and development of beneficial microbes. In addition, numerous studies reported the capacity of biochar to suppress plant diseases (reviewed by [22]), although studies about phytoparasitic nematodes are lacking.

The use of solid olive wastes to make biochars for agricultural production enters well in the initiatives of the European Waste Framework Directive (Directive 2008/98/EC, November 19, 2008). More recently, the movement towards the green economy and better governance (EC COM 363 of June 20, 2011) considers the development of a growing economy to improve human well-being and environmental sustainability. Specifically, an ideal economic model incorporates the circular economy, with the goal to reduce the consumption of resources and the creation of waste; through the management, reuse and recycling of wastes generated during the production, distribution and consumption processes. In order to understand how biochars from recycled OMW can improve conditions in the agro-ecosystem, investigations need to identify the factors that determine the positive and negative characteristics of this organic product on the different biological components.

Previous studies by [14] examined the effects of pyrolysis temperature (PT) on the chemical properties and phytotoxic activity of biochar produced from three-phase OMW. Moreover, the ability of OMW biochar to remediate soils contaminated with metals was also analyzed [8, 23]. However, to date, very few results clearly explain the relationship between chemical composition of OMW biochars and their biological activity [24]. In the current work, the effects of various PTs on the chemical composition and morphological features of biochars produced from two-phase solid OMW were evaluated. In order to elucidate the chemical and structural basis of the effects of these biochars on different selected organisms, OMW and the char products were characterized and compared by using multiple techniques: solid state ^13^C magic angle spinning (MAS) nuclear magnetic resonance (NMR) spectroscopy, liquid chromatography-electrospray ionization-time of flight-mass spectrometry (LC-ESI-TOF-MS), and scanning electron microscopy (SEM). The combined use of ^13^C MAS NMR and LC-ESI-TOF-MS allows a complementary chemical characterization of OMW. In this regard, the use of ^13^C MAS NMR spectroscopy allows detailed monitoring of the chemical changes of the main C types occurring in plant biomass following pyrolysis [25, 26], providing information on the entire complex rather than just the single, individual molecules [27]. Conversely, LC-ESI-TOF-MS can be used to detect and identify an elevated number of metabolites and presents higher sensitivity than NMR spectroscopy [28].

The organic feedstock was treated at four different PTs (ranging from 300 °C up to 1000 °C), then the effect of the derived water extracts was tested using a multi-species bioassay approach. This study, for the first time, investigated simultaneously the association between the composition of OMW or its biochar and the growth of two higher plants, four fungi and one nematode species. Among soil organisms, nematodes are considered good bioindicators in environmental monitoring, as their population is sensitive to pollutants and environmental perturbations [29]. The analysis of nematode community composition provides useful information on the effects of agricultural practices and soil nutrient status, thus affording rapid decisions for management activities and remediation [30]. Specifically, our work aims to investigate:

1. the effect of different PTs on the physicochemical and morphological features of biochars obtained from two-phase solid OMW;
2. the effect of water extracts of untreated and pyrolyzed OMW on the growth of different plant, nematode and fungal species;
3. the association of OMW and biochar chemistry, as defined by ^13^C MAS NMR spectroscopy, LC-ESI-TOF-MS and elemental composition, to the growth performance of the selected species.

## Materials and methods

### Olive waste material and thermal treatments

Solid OMW obtained from a two-phase extraction system, at an olive oil facility located in the province of Vibo Valentia (Italy), was used as feedstock. The waste had a composition of: 54.6% C, 1.60% N, C/N ratio 35.3, H/C ratio 1.66, pH 5.78 and EC 1067 μs/cm. The organic material had an initial water content of ~20%. It was air dried at room temperature in a ventilated chamber until a constant weight was obtained (approximately after 30 days), and then stored in sealed containers at room temperature. Subsequently, 50 g of the dried samples were subjected to thermal treatments at 4 different PTs (300 °C, 500 °C, 800 °C and 1000 °C) for 5 h in a muffle furnace. The organic samples were enwrapped in aluminum foils to ensure a very low oxygen availability during the thermal treatment. Untreated samples served as controls. Each treatment was replicated three times. Samples were homogenized in a blender to a particle size <2 mm, then the material was placed in air-tight containers and stored until use.

### Characterization of organic materials

#### Elemental analyses, pH and electrical conductivity

The elemental C, N and H of the organic materials were determined by flash combustion of micro samples (5 mg) in an Elemental Analyzer NA 1500 (Fison 1108 Elemental Analyzer, Thermo Fisher Scientific). The pH was determined by using a pH-meter (Basic 20 CRISON, Barcelona, Spain), and electrical conductivity (EC) was measured with a CRISON conductometer, in suspensions of 1:2.5 and 1:5 organic material:water, respectively.

#### ^13^C MAS NMR

The organic samples produced at different PTs were analyzed by solid state ^13^C NMR spectroscopy, in order to conduct a semi-quantitative evaluation. All measurements were performed on a Bruker AV-300 NMR spectrometer (Bruker, Karlsruhe, Germany), equipped with a 4 mm wide-bore MAS probe. Samples (60–80 mg) were packed into 4 mm zirconia rotors coupled with Kel-F caps and spun at a rate of 10,000 ± 2 Hz. The Cross Polarization (CP) MAS NMR experiment was performed by using the following acquisition parameters: 1814 time domain points, a spectral width of 300 ppm (22727.3 Hz), 2 s recycle time, 1 ms contact time, 20 ms acquisition time, and 4000 scans. The CPMAS pulse sequence also included a ^1^H ramp to account for non-homogeneity of the Hartmann-Hahn condition developed at high rotor spin rates. For the Direct Polarization (DP) MAS NMR experiment, it was necessary to acquire a ^13^C NMR spectrum of biochar obtained at 1000 °C due to the extremely low content of CP-donor hydrogens, making ineffective the CPMAS acquisition (spectrum not shown). ^13^C DPMAS spectrum was obtained by setting the following acquisition parameters: 1814 time domain points, a spectral width of 300 ppm (22727.3 Hz), 30 s recycle time, 20 ms acquisition time, and 4000 scans. In all cases, carbon-proton heteronuclear coupling was suppressed by applying a TPPM-15 (Two-Pulse Phase Modulation) decoupling scheme.

Each ^13^C MAS NMR spectrum was manually divided into discrete regions which were selected and assigned according to previous reference studies [22, 31]: 0–45 ppm = alkyl C; 46–60 ppm = methoxyl and N-alkyl C; 61–90 ppm = O-alkyl C; 91–110 ppm = di-O-alkyl C; 111–140 ppm = H- and C-substituted aromatic C; 141–160 ppm O-substituted aromatic C (phenolic and O-aryl C); and 161–190 ppm carboxyl C. For each spectrum, the areas under the curve range, within these spectral regions, were integrated and normalized.

#### LC-MS

The extraction of polyphenols from OMW and its biochars was performed according to the procedure described by [32], with some modifications. Briefly, 0.5 g of dried samples were suspended in 10 ml of a water-methanol solution (H_2_O:MeOH = 20:80), sonicated for 10 min in an ice bath and centrifuged at 4000 rpm, 4 °C for 10 min. The supernatants were collected, dried in a speed-vac (Savant, Thermo, USA) and re-suspended in 2 ml of H_2_O:MeOH solution (50:50). Samples were then filtered (0.22 μm) and stored at -20 °C in the dark until use.

All analyses were conducted on an Agilent HP 1260 Infinity Series liquid chromatograph equipped with a DAD system (Agilent Technologies, Santa Clara, CA, USA) coupled to a Q-TOF mass spectrometer model G6540B (Agilent Technologies). Separations were performed on a ZorbaxEclips Plus C18 column, 4.6 x 100 mm, with 3.5 μm particles (Agilent Technologies). The analyses were performed at a constant temperature of 37 °C, using a linear gradient system composed of 0.1% (v/v) formic acid in water (phase A), and 0.1% (v/v) formic acid in acetonitrile (phase B). The flow was 0.6 ml min^-1^, 95% A graduating to 100% B in 12 min, 100% B 12–15 min, 95% A 15–17 min and equilibrating at 95% A 17–20 min. The UV spectra were collected by DAD every 0.4 s from 190 to 750 nm with a resolution of 2 nm. The MS system was equipped with a Dual Electrospray Ionization (ESI) source and operated with Agilent MassHunter Data Acquisition Software, rev. B.05.01 in the negative mode. Mass spectra were recorded in the range *m/z* 100–1600 as centroid spectra, with 3 scans per second. Two reference mass compounds were used to perform the real-time lock mass correction, purine (C_5_H_4_N_4_ at *m/z* 121.050873, 10 μmol L^−1^) and hexakis (1H,1H, 3H-tetrafluoropentoxy)-phosphazene (C_18_H_18_O_6_N_3_P_3_F_24_ at *m/z* 922.009798, 2 μmol L^−1^). The capillary was maintained at 4000 V, Fragmentor at 180 V, cone 1 (skimmer 1) at 45 V. Gas temperature was 350 °C during the run at 11 L min^-1^, and the nebulizer was set at 45 psig. The injected sample volume was 5 μL. Solvents were LC–MS grade, and all other chemicals were analytical grade (from Sigma-Aldrich, Germany, unless otherwise stated; ESI–TOF tune mix from Agilent Technologies).

Data were evaluated using MassHunter Qualitative Analysis Software B.06.00 and comparisons were made to known compounds in an in-house database combined with data from the literature. Positive identifications of plant metabolites were considered for analysis if the compound was detected with a mass error below 10 ppm and with a sufficient score.

### Morphological characterization

The characterization of biochar morphology was performed using a scanning electron microscope (SEM ZEISS EV040). Samples were dehydrated in ascending concentration of alcohol under a laminar flow hood; subsequently dried in a critical point driver (EMITECH K850), then sputter coated with gold-palladium (AGAR sputter COATED B 7340). The SEM observations of all samples were performed at magnifications of 1K, 1.6K or 2.4K, with an accelerating voltage of 20.00 kV, at 10 mA current and 15.5 mm < WD < 16.5 mm as a focal length. Several specimen pots were prepared and observed for each sample in order to analyse the microstructure variations.

### Plant and fungal bioassays

A seed germination experiment was carried out to evaluate the effects of the olive waste and four biochars on the growth of two plant species. Garden cress (*Lepidium sativum* L.) and field mustard (*Brassica rapa* L. subsp. *oleifera*) were selected as the target species because of their well-known sensitivity to phytotoxic compounds and for agro-economic importance, respectively. Water extracts of both OMW and biochars were obtained by adding the dried, powdered samples to water (50 g L^-1^), leaving in orbital agitation for 5 h at room temperature. The suspensions were centrifuged (2395 *g* for 10 min), filter-sterilized (0.22 μm), then stored at −20 °C until use. *In vitro* experiments were carried out in Petri dishes (90 mm diameter) containing a sterile filter paper disks (Whatman Grade 1) wetted with 4 ml of each water extract treatment or water as a control. Seeds were placed in a growth chamber at 24 °C in the dark. The seedling root length was measured at 36 hours for *L*. *sativum*, and at five days for *B*. *rapa* after seeding. Five biological replicates were used for each treatment, each consisting of 25 seeds for each plant species.

Similarly, we evaluated the effects of water extracts obtained from biochars on the growth of four different filamentous fungi: *Aspergillus niger*, *Fusarium oxysporum*, *Rhizoctonia solani* (three plant pathogens), and *Trichoderma harzianum* (a saprophyte and a plant beneficial microbe). The strains were obtained from the mycology laboratory of the Department of Agricultural Sciences at the University of Naples Federico II (Italy), and maintained on potato dextrose agar (PDA, Oxoid) medium. Water extracts were tested alone or in presence of potato dextrose broth (PDB, Oxoid; that served as an external source of organic carbon and nutrients) to evaluate the inhibition of fungal growth by the biochar constituents, i.e. presence of toxic compounds, or to the lack of suitable carbon sources.

Fungal colonies were inoculated to 1% (w/v) water agar (WA, Oxoid) plates and incubated at 25 °C. After 7 days, 4 mm diameter fungal plugs, were transferred to Petri dishes containing 5 ml of WA amended with 5 ml of each filter-sterilized OMW or biochar extract. The cultures were incubated at 24 °C in the dark. After 72 h hyphal density was measured by observation under an optical microscope at five randomly chosen points, by determining under the number of hyphae crossing a 1 mm line at 250 × magnification, and the radial growth of each colony. A growth index was calculated as the product of the fungal colony area, calculated from the measured colony radius, and the hyphal density, according to [33]. PDA plates were used as controls and each treatment was replicated 10 times.

### Nematode bioassay

The effect of water extracts of OMW and its biochars on the survival of the root-knot nematode *Meloidogyne incognita* (Kofoid and White) Chitwood, was evaluated *in vitro* in controlled conditions, as previously reported [34, 35]. Briefly, eggs of *M*. *incognita* were collected from infested roots of tomato (*Solanum lycopersicum* L. cv. Roma), using NaOCl [36]. Second-stage juveniles (J2s) were collected from eggs spread in hatching boxes using a modified Jenkins method [37]. Thirty freshly hatched (24h old) *M*. *incognita* J2s were added to individual wells, containing 1 ml of each water extract. Each well was sealed with Parafilm (Sigma Aldrich) and incubated at 25 ± 2 °C in the dark. Water-exposed J2s were used as controls. Nematode mobility was evaluated every 24h for a total of 15 days. The experiment was repeated twice and each treatment consisted of four replicates.

### Data analysis

Data from bioassays were normalized to controls and subjected to statistical analysis (two-way ANOVA) using the STATISTICA 7 software (StatSoft Inc., Tulsa, OK, USA). For each bioassay data analyses were separately performed on the nematode, plant species and fungi, with or without the addition of the external carbon source. Pairwise differences were tested using Tukey’s HSD post-hoc test. One-way ANOVA was used to assess the effect of PT (5 levels) on biochar chemistry as assessed by chemical analyses and ^13^C NMR spectroscopy.

The association between species performance in the bioassays and organic feedstock and biochar biochemistry was measured by calculating linear correlation between species growth on the water extracts (50 g L^-1^ of OMW and the biochar pyrolyzed at 300 °C, 500 °C, 800 °C, and 1000 °C) and the ^13^C NMR data, as well as with basic chemical parameters (i.e. pH, EC, C and N content, C/N and H/C ratios) of the same test material. Species performance was evaluated for correlations to the ^13^C MAS NMR spectral regions (N = 7) selected from reference literature [31, 38]. In order to control for multiple comparisons, the correlation was tested for statistical significance at α = 0.05/N, with N being the number of performed correlation tests, by applying the Bonferroni’s correction.

## Results

### Effect of thermal treatments on composition and structure of organic materials

Thermal treatments for 5 h at different temperatures (300 °C, 500 °C, 800 °C or 1000 °C) produced significant differences in element composition, pH and EC of the organic materials (Table 1). Although the carbon content increased from 54.6% to 77.9%, no statistical differences were found among the biochars pyrolyzed at 500 °C, 800 °C or 1000 °C. Generally, increased PT corresponded to higher values of C content, C/N ratio, pH and EC. Conversely, a progressive decrease in N content and H/C ratio was observed as PT increased. These differences were particularly evident in the biochar treated at 500 °C when compared to the untreated OMW (control) or to biochar from PT of 300 °C (Table 1).

**Table 1:** Chemical characteristics of olive mill waste (OMW) and its biochars pyrolyzed at 300 °C, 500 °C, 800 °C, or 1000 °C for carbon and nitrogen contents, C/N and H/C ratios, pH, and electric conductivity (EC).

	C %	N %	C/N	H/C	pH	EC μs/cm
**OMW**	54.6 c	1.60 a	35.3 d	1.66 a	5.78 d	1067 c
**Biochar 300 °C**	68.1 b	1.13 b	60.2 c	1.12 b	8.53 c	820 c
**Biochar 500 °C**	72.7 ab	0.37 c	217.1 b	0.19 c	8.98 c	5062 b
**Biochar 800 °C**	77.9 a	0.34 c	210.5 b	0.12 d	9.62 b	8326 a
**Biochar 1000 °C**	76.2 a	0.11 d	762.4 a	0.02 e	10.68 a	8675 a

Different letters within each column indicate significant differences by the Tukey test at *p* < 0.05.

**Fig 1:**
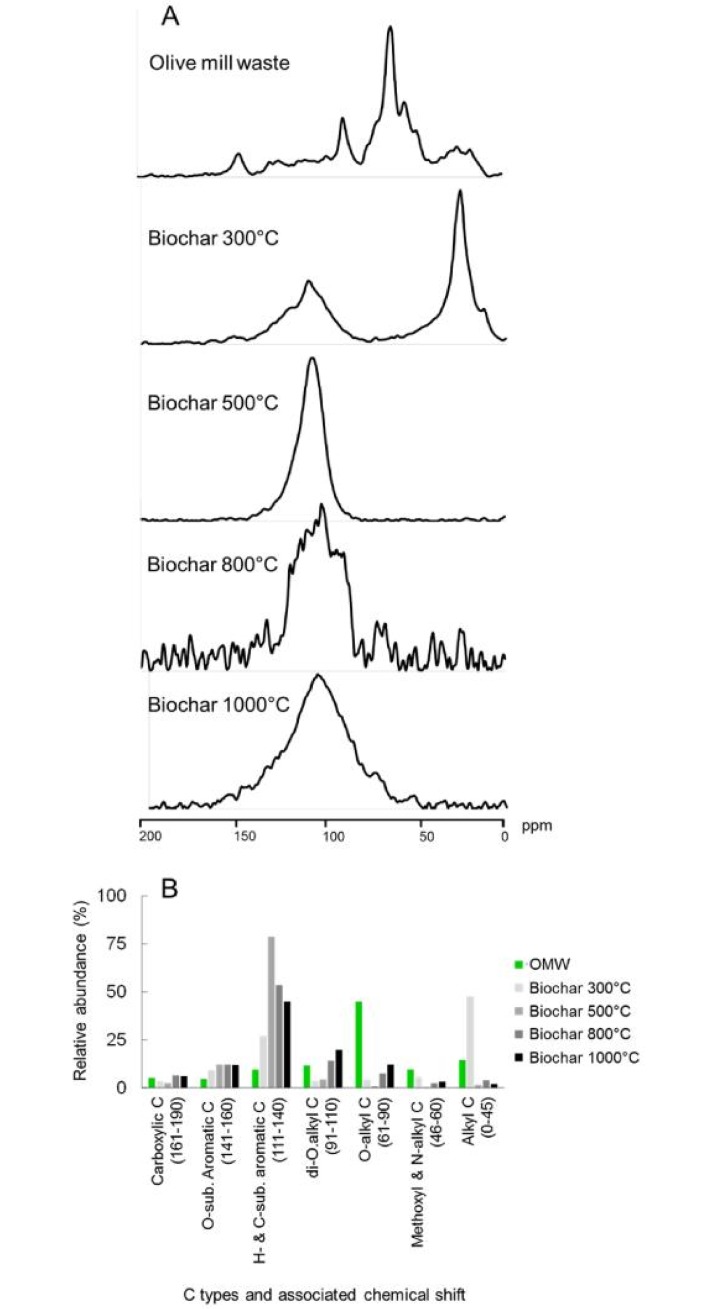
Characterization of the water extracts from olive mill waste (OMW) and its biochars pyrolyzed at 300 °C, 500 °C, 800 °C, or 1000 °C for 5 hours. (A) ^13^C-CPMAS NMR spectra; (B) relative abundance (%) of the seven main classes of organic C types corresponding to the spectra assessed by ^13^C-CPMAS NMR spectroscopy.

Solid state ^13^C MAS NMR spectroscopy revealed significant differences in spectra profiles of the organic materials (Fig 1). In comparison to pyrolyzed materials, the OMW spectrum was particularly rich in O-alkyl C (61–90 ppm) and di-O-alkyl-C (91–110 ppm) fractions, which are often associated with sugars and polysaccharides (Fig 1A). The heat treatment at 300 °C determined an increase of components in the aliphatic alkyl-C region (0–45 ppm). Higher PTs augmented the relative abundance of the aromatic regions (111–140 ppm and 141–160 ppm). This was particularly evident in the biochar pyrolyzed at 500 °C, where the H- and C-substituted aromatic C fraction (111–140 ppm) represented more than 75% of the total abundance of organic C (Fig 1B).

**Fig 2:**
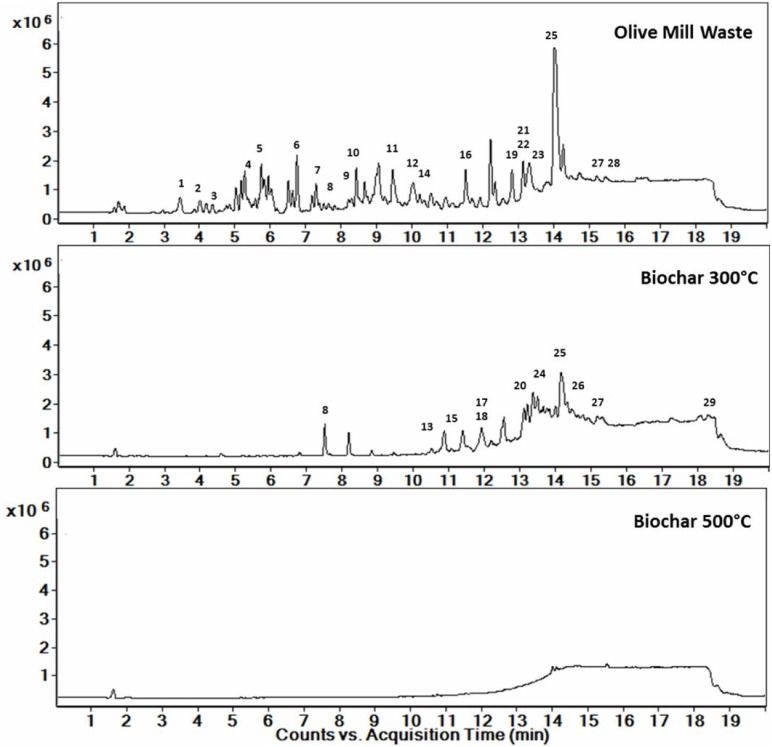
Total ion chromatograms (TIC) of olive mill waste and its biochars pyrolyzed at 300 °C and 500 °C as obtained by LC-MS.

Chromatographic profiles obtained by LC-MS analysis also confirmed that the thermal treatments modified the composition of the organic materials in the OMW and biochars (Fig 2). A notable reduction of the number of compounds detected and the quantity/area under the peaks that was observed, that was inversely proportional to the increased PT. The chromatograms of biochars pyrolyzed at higher temperatures (500 °C, 800 °C and 1000 °C) were similar, with no significant signals observed (Fig 2C). Conversely, in OMW and biochar 300 °C, numerous peaks were separated (Fig 2A and 2B, respectively), and the UV and mass spectra were analysed to provide the putative identification of up to 29 compounds (Table 2). These included fatty acids (peaks 3, 10, 11, 12, 13, 15, 16, 18, 19, 20, 21, 23, 24, 26, 27, 29; Table 2), dicarboxylic acids (peaks 6, 8, 17, 25), carboxylic acid derivatives (peaks 1, 2), phenolic compounds (peaks 4, 5, 7, 9) and triterpene acids (peaks 14, 22, 28). Some of these compounds were found both in OMW and biochar 300 °C (peaks 8, 25, 27), but the majority were exclusively present in OMW or in biochar 300 °C (Table 2). The pyrolysis at 300 °C determined the decrease in the abundance of 2-hydroxy stearic acid (peak 25), which represented the most abundant compound in both extracts (Fig 2, Table 2). In parallel, an increase of the carboxylic acid derivatives nonic acid (peak 8) and 9,10-Epoxystearic acid (peak 27) were observed. However, in OMW the majority of the peaks with retention times of 3–10 min (including triterpene acids and phenolic compounds) almost disappeared when the organic material was thermally processed, in favour of dicarboxylic and fatty acids.

**Table 2:** Compounds identified in olive mill waste (OMW) and its biochar pyrolyzed at 300 °C (300 °C) as analyzed by LC-MS. Data includes retention time (RT), experimental and calculated masses (based on the chromatograms in the run and on the formula, respectively), description of compound, chemical formula, similarity score, family, sample type and peak area as total volume (as percentage in parenthesis). Peak numbers have been shown also in Fig 2.

Peak No.	RT (min)	Mass exp.	Description	Formula	Mass calc.	Score	Family	Sample Type	Peak Area—Volume (%)
**1**	3.453	232.0962	Glycerol-1-Propanoate diacetate	C_10_H_16_O_6_	232.0947	79	Carboxylic acid derivative	OMW	4207838 (1.69%)
**2**	3.99	370.1618	4-Hydroxy-3,5-bis(1-methylethyl)phenyl glucuronide	C_18_H_26_O_8_	370.1628	80	Carboxylic acid derivative	OMW	2546185 (1.03%)
**3**	4.376	172.0748	2-Octenedioic acid	C_8_H_12_O_4_	172.0735	80	Fatty acid	OMW	1526124 (0.61%)
**4**	5.457	390.1176	Oleoside	C_16_H_22_O_11_	390.3410	79	Phenolic compound	OMW	191433 (0.08%)
**5**	5.745	450.1519	Auriculoside	C_22_H_26_O_10_	450.1530	96	Phenolic compound	OMW	4635493 (1.87%)
**6**	6.755	186.0905	5-Butyltetrahydro-2-oxo-3-furancarboxilic acid	C_9_H_14_O_4_	186.0892	92	Dicarboxylic acid	OMW	8292435 (3.34%)
**7**	7.281	540.1855	Oleuropein	C_25_H_32_O_13_	540.1843	99	Phenolic compound	OMW	4184655 (1.68%)
**8**	7.515	188.1062	Nonic acid	C_9_H_16_O_4_	188.1049	79	Dicarboxylic acid	OMW	2833930 (0.63%)
								300 °C	4029244 (2.02%)
**9**	8.21	286.0498	Luteolin	C_15_H_10_O_6_	286.0477	97	Phenolic compound	OMW	1951498 (0.79%)
**10**	8.307	188.1427	3-Hydroxydecanoic acid	C_10_H_20_O_3_	188.1412	77	Fatty acid	OMW	2833930 (1.14%)
**11**	9.455	332.2593	9,10,13-Trihydroxystearic acid	C_18_H_36_O_5_	332.2562	79	Fatty acid	OMW	13027513 (5.24%)
**12**	10.018	330.2433	(11E)-9,10,13-Trihydroxyoctadec-11-enoic acid	C_18_H_34_O_5_	330.2406	73	Fatty acid	OMW	11372913 (4.58%)
**13**	10.515	328.2264	9-hydroperoxy-12,13-epoxy-10-octadecenoic acid	C_18_H_32_O_5_	328.2250	85	Fatty acid	300 °C	1650154 (0.83%)
**14**	10.672	488.3514	Rotundic acid	C_30_H_48_O5	488.3502	95	Triterpene acid	OMW	3268305 (1.32%)
**15**	11.085	292.205	12,13S-epoxy-9Z,11,15Z-octadecatrienoic acid	C_18_H_28_O_3_	292.4131	81	Fatty acid	300 °C	1105535 (0.55%)
**16**	11.516	314.2484	12,13-dihydroxyoctadec-9-enoic acid	C_18_H_34_O_4_	314.2457	89	Fatty acid	OMW	10746380 (4.33%)
**17**	11.93	286.2169	Hexadecanedioic acid	C_16_H_30_O_4_	286.2144	92	Dicarboxylic acid	300 °C	4069764 (2.04%)
**18**	11.978	298.2165	9-hydroxy-12Z-octadecenoic acid	C_18_H_34_O_3_	298.2508	96	Fatty acid	300 °C	4069764 (2.04%)
**19**	12.824	296.2366	(9*Z*,11*E*,13*S*)-13-hydroxyoctadeca-9,11-dienoic acid	C_18_H_32_O_3_	296.2351	94	Fatty acid	OMW	10325521 (4.16%)
**20**	13.117	326.2476	8-methoxy-13-hydroxy-9,11-octadecadienoic acid	C_19_H_34_O_4_	326.2457	88	Fatty acid	300 °C	6144283 (3.08%)
**21**	13.136	294.2218	(10*E*,12*Z*)-9-oxooctadeca-10,12-dienoic acid	C_18_H_30_O_3_	294.2195	99	Fatty acid	OMW	2396396 (0.96%)
**22**	13.174	472.3564	Maslinic Acid	C_30_H_48_O_4_	472.3553	99	Triterpene acid	OMW	2396396 (0.96%)
**23**	13.254	298.2524	cis-9,10-Epoxystearic acid	C_18_H_34_O_3_	298.2508	98	Fatty acid	OMW	4245475 (1.71%)
**24**	13.662	340.2632	15S-hydroperoxy-11Z,13E-eicosadienoic acid	C_20_H_36_O_4_	340.2614	91	Fatty acid	300 °C	5163517 (2.58%)
**25**	14.016	300.2681	2-hydroxy stearic acid	C_18_H_36_O_3_	300.2664	91	Dicarboxylic acid	OMW	38695536 (15.58%)
								300 °C	7148757 (3.58%)
**26**	14.142	342.2788	Eicosanedioic acid	C_20_H_38_O_4_	342.2770	91	Fatty acid	300 °C	20364018 (10.19%)
**27**	15.205	298.2518	9,10-Epoxystearic acid	C_18_H_34_O_3_	298.2508	98	Fatty acid	OMW	703598 (0.28%)
								300°C	3479100 (1.74%)
**28**	15.529	456.3612	Oleanolic acid	C_30_H_48_O_3_	456.3603	98	Triterpene acid	OMW	738560 (0.30%)
**29**	18.306	426.3723	Hexacosanedioic acid	C_26_H_50_O_4_	426.3709	95	Fatty acid	300 °C	26177528 (13.1%)

**Fig 3:**
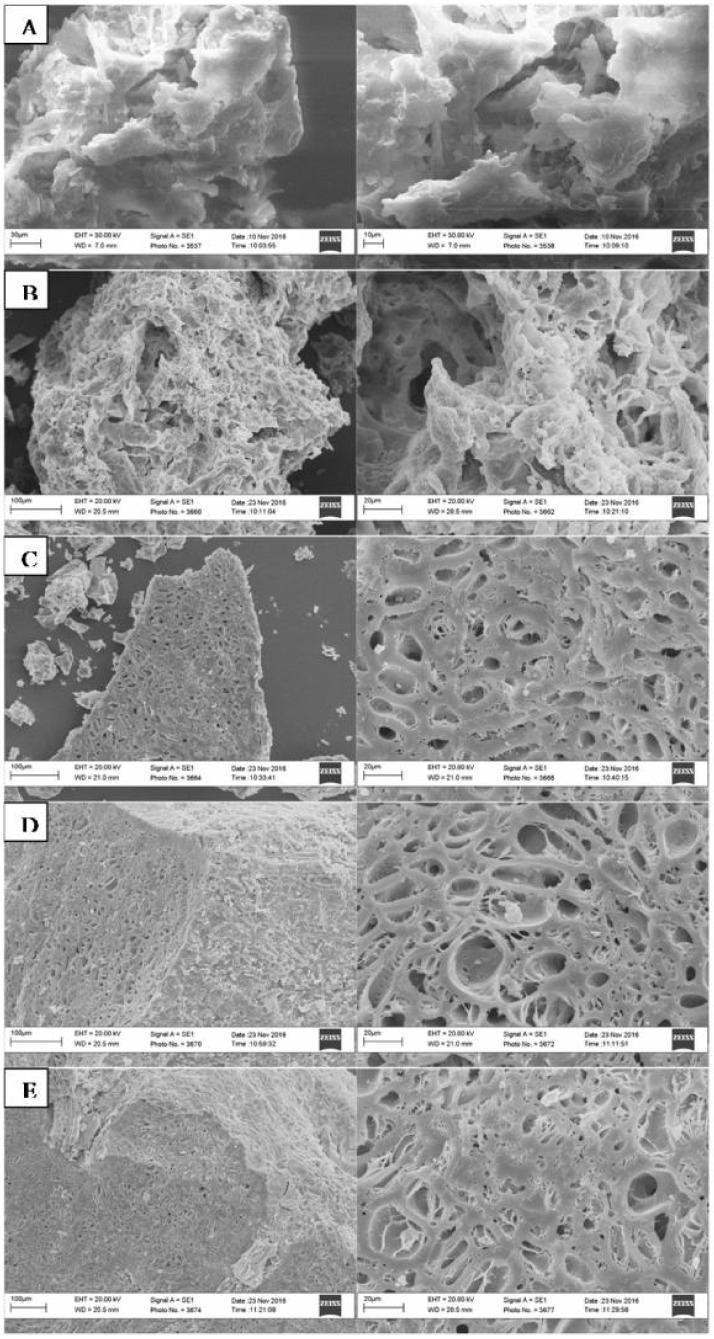
Scanning electron microscope (SEM) images of: (A) olive mill waste (OMW); and its biochars pyrolyzed at (B) 300 °C, (C) 500 °C, (D) 800 °C, and (E) 1000 °C for 5 hours. External morphology of the organic material magnified at 1.01 K times in the left column, and at higher magnification of 2.00K of the same on the right side.

Microscopic observations of OMW and the biochars produced at the four different PTs were performed by scanning electron microscopy (SEM). The images revealed that the temperatures of the treatment affected the biochar external morphology (Fig 3). This effect was particularly evident when comparing the biochar structural conformation with OMW. The untreated material showed a spongy appearance with numerous hollows, while the samples obtained at 300 °C exhibited an increased porosity, possibly related to the decreased abundance of sugars and polysaccharides observed in the ^13^C NMR spectra. As the PT increased (500 °C, 800 °C and 1000 °C), the biochar surfaces appeared smoother, with the development of uneven pores of various diameter (ranging between 0.1 and 10 μm).

### Effects of biochar water extracts on plants and fungi

**Fig 4:**
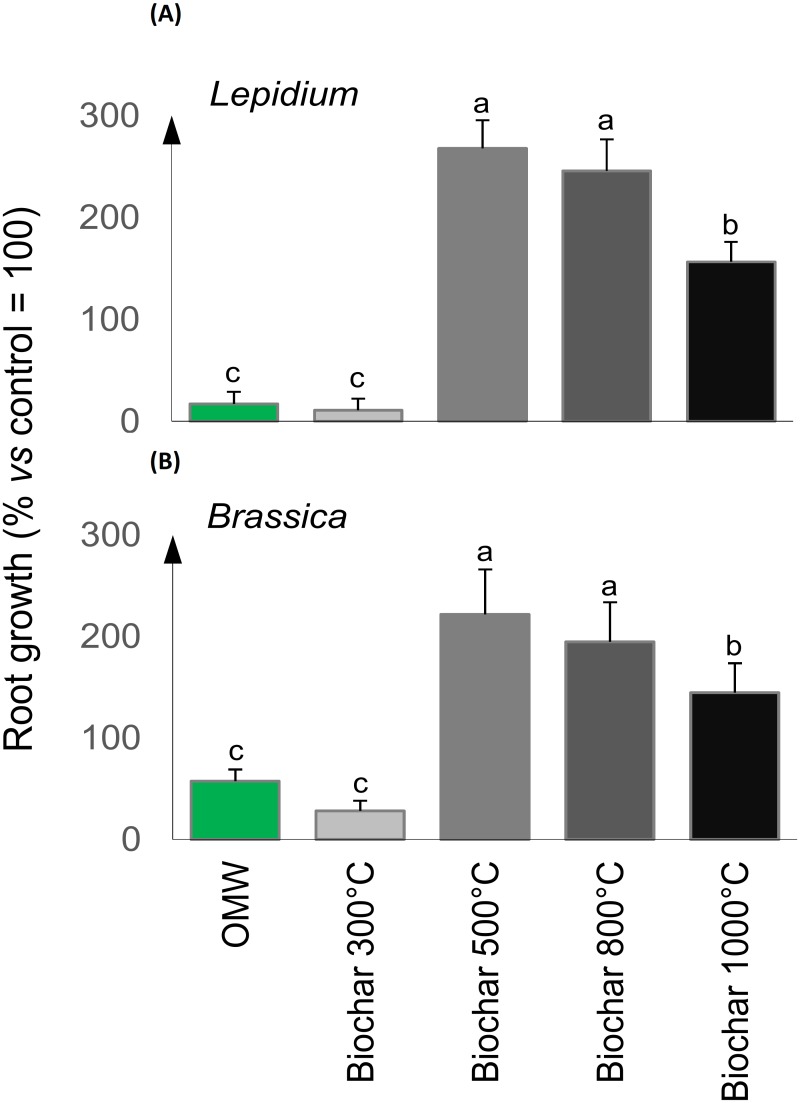
Root growth responses of (A) *Lepidium sativum* and (B) *Brassica rapa* to water extracts (50 g L^-1^) of olive mill waste (OMW) and its biochars pyrolyzed at 300 °C, 500 °C, 800 °C, or 1000 °C for 5 hours. The growth of treated roots were normalized to those of the water-treated plants (controls). Different letters indicate significant differences by the Tukey test at *p*<0.05.

Experiments on the effects of the different biochar water extracts on plant germination and root growth showed similar results for *L*. *sativum* and *B*. *rapa* (Fig 4). There was greater inhibition, that was significantly different, shown for the root length of plants treated with water extracts from OMW or biochar pyrolyzed at 300 °C in comparison to those obtained from biochars treated at higher temperatures. A consistent reduction in the initial phytotoxicity was observed for biochar water extracts produced at 500 °C and 800 °C (Fig 4).

**Fig 5:**
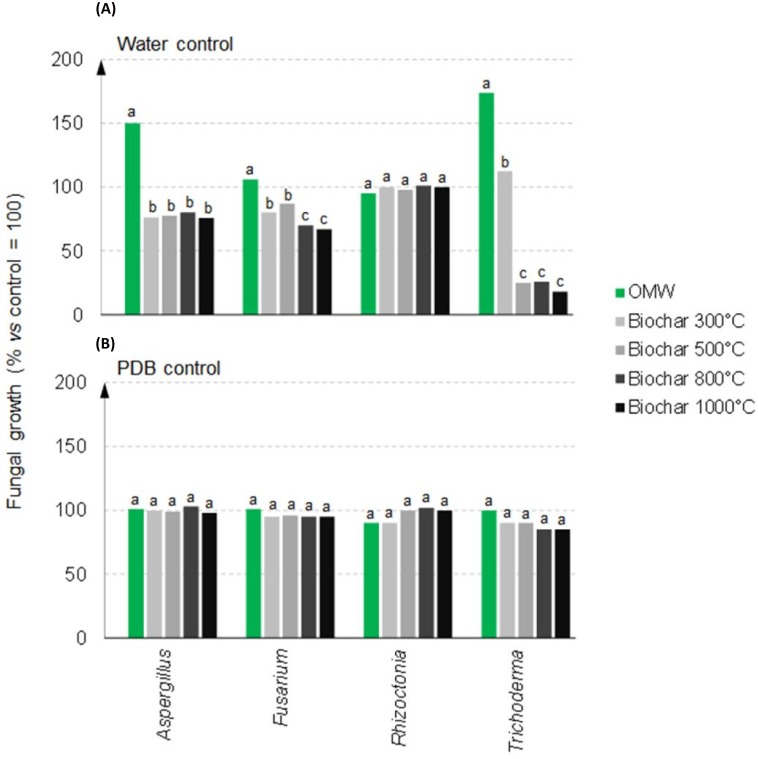
*In vitro* mycelial growth responses of four fungi: *Aspergillus niger*, *Fusarium oxysporum*, *Rhizoctonia solani* (three plant pathogens), and *Trichoderma harzianum* (beneficial microbe) to water extracts (50 g L^-1^) of olive mill waste and its biochars pyrolyzed at 300 °C, 500 °C, 800°C, or 1000 °C for 5 hours. The extracts were tested (A) alone, or (B) in the presence of potato dextrose broth (PDB), that served as an external source of organic carbon and nutrients. Fungal growth of the treatments were normalized to those of the control fungal cultures grown on PDA alone. Different letters within each graph indicate significant differences by the Tukey test at *p*<0.05.

The biological effects of the organic feedstock and its biochars were also tested on the growth of different fungal species (Fig 5). The results indicated that water extracts from all treatments affected fungal growth when tested alone, but no significant effects were observed in the presence of PDB as nutrient supplement, when compared to the control (Fig 5). Interestingly, both *Aspergillus* and *Trichoderma* grew much better in plates amended with OMW extract, which was significantly different than growth noted in the presence of all biochar water extracts. In particular, the water extracts of biochars produced at PTs ≥ 500 °C significantly inhibited *Trichoderma* in comparison to those at PT = 300 °C. Inhibition was similarly observed for *Fusarium* treated with the biochar water extracts, but this variation was relatively less than that observed with the previous two fungi. However, the growth of *Rhizoctonia* was diverse in that no differences were found between the extracts from OMW feedstock and all pyrolyzed materials (Fig 5).

### Activity of biochars on nematodes

**Fig 6:**
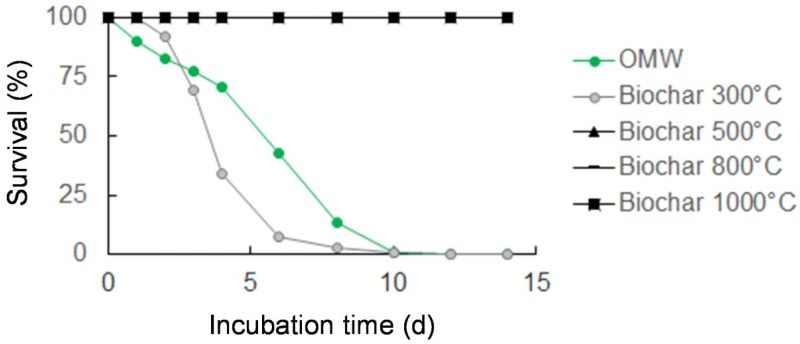
Effects of water extracts (50 g L^-1^) from olive mill waste (OMW) and biochars produced at PT 300 °C, 500 °C, 800 °C, or 1000 °C for 5 hours on the survival of the root-knot nematode *Meloidogyne incognita*. The survival data from treatments with biochars at PT 500 °C, 800 °C and 1000 °C are similar, thus the points overlap and are represented only by symbols for biochar 1000 °C in the graph.

The effects of water extracts from OMW and its pyrolyzed materials on the survival of the root-knot nematode *M*. *incognita* were evaluated *in vitro*. Results indicated that the extract from the organic feedstock completely immobilized the nematodes after 10 days of incubation (Fig 6). Similar results were also observed with biochar water extract produced at 300 °C over the time period, although the reduction in survivability was more rapid, demonstrating a 50% decline in nematode survival only after 3.5 days. In contrast, no detrimental effects were observed on *M*. *incognita* survival (100%) treated with the biochar water extract from PT 500 °C, 800 °C or 1000 °C.

### Relationship between biological parameters and chemical properties of organic materials

**Fig 7:**
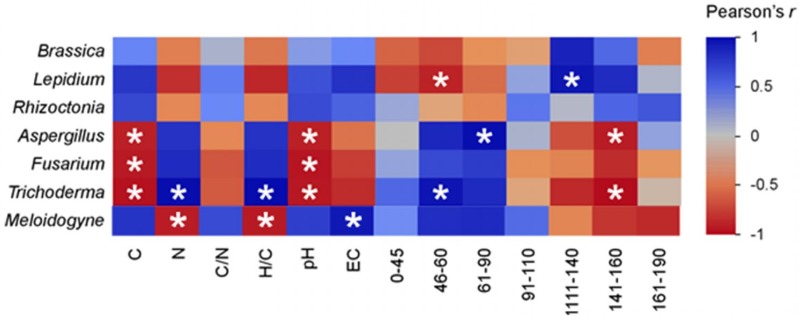
Heat-map reporting the correlations (Pearson’s r) of the growth on water extracts of six target organisms: Two plants (*Brassica*, *Lepidium*), four fungi (*Rhizoctonia*, *Aspergillus*, *Fusarium*, *Trichoderma*) and survival of a nematode (*Meloidogyne*), to the chemical parameters of olive mill waste (OMW) and its biochars pyrolyzed at 300 °C, 500 °C, 800 °C, or 1000 °C. Asterisks indicate statistically significant correlation values, negative or positive. Labels in the *x*-axis indicate the parameters measured (carbon and nitrogen contents, pH, C/N and H/C ratios, EC), and the numbers refer to the ^13^C NMR spectral ranges (in ppm) for reference regions of the different C types: alkyl C (0–45 ppm), methoxyl and N-alkyl C (46–60 ppm), O-alkyl C (61–90 ppm), di-O-alkyl C (91–110 ppm), H- and C-substituted aromatic C (111–140 ppm), O-substituted aromatic C (141–160 ppm), carboxylic C (161–190 ppm).

Fig 7 reports the correlations of the plant, fungal and nematode growth responses to the water extracts of feedstock and biochar and their chemical properties, as determined by chemical analyses and ^13^C NMR spectral signals. In general, the correlation patterns among the individuals within each biological group, plants and fungi, were similar for many parameters. The fungal pathogen *Rhizoctonia* was an exception, demonstrating more correspondence to the plants instead of the other fungi. Opposite responses were also noted between the plant group and the microbe group for relationships relative to growth with the OMW and biochar characters. Specifically, C content, pH, EC and the spectral region of O-substituted aromatic C at 141–160 ppm were negatively correlated to *Aspergillus*, *Fusarium* and *Trichoderma* fungal growth (Fig 7). Conversely, significant positive correlations were found between plant or *Rhizoctonia* growth and N content, H/C ratio and relative abundance of the two regions related to the aromatic C (111–140 and 141–160 ppm). In the case of *M*. *incognita*, the heat map revealed similar correlations to those observed for *L*. *sativum* growth in terms of elemental composition or chemical parameters, but an opposite trend in the spectral signals. In particular, nematode survival was found to be positively correlated only to the resonance regions of alkyl C (0–45 ppm), methoxyl and N-alkyl C (46–60 ppm), O-alkyl C (61–90 ppm), and di-O-alkyl C (91–110 ppm). No significant correlations were observed between the responses of the tested organisms to the C/N ratio or the spectral regions of the alkyl C (0–45 ppm), the di-O-alkyl C (91–110 ppm) or the carboxylic C (161–190 ppm).

## Discussion

OMW subjected to 4 different thermal treatments (temperatures between 300 °C to 1000 °C) produced biochars showing numerous differences in chemical composition and morphology compared to the original organic feedstock (Table 1, Figs 1–3). C content and C/N ratio, pH and EC increased remarkably in parallel to temperature, while an opposite trend was observed for N content and H/C ratio. These findings are consistent with those in previous studies performed on feedstocks from various sources [13, 15, 39, 40]. The decrease of N content has been related to the production of ammonia and other volatile compounds containing N during pyrolysis [41, 42]. The decrease of the H/C molar ratio suggested that the biochars became more aromatic and carbonaceous at higher values of PT. In fact, the H/C ratio of OMW was 1.60, that corresponded to values reported for unburned materials such as cellulose or lignin, whereas H/C values ≤ 0.2 found in biochars produced at 500 °C and 800 °C could be attributed to those of “black carbon” [43]. An H/C ratio ≤0.1, as observed in biochar obtained at 1000 °C, may indicate a graphite-like structure [39]. In addition, all the biochars showed an alkaline pH, ranging from 8.53 to 10.68. High pH values may be responsible for the increase in soil pH observed after making biochar amendments [44], that contributes to negative effects in sandy soils having a low-buffering capacity [41]. Biochar EC values showed a continuous increase associated to the enhanced temperatures of the thermal treatments that may be related to the loss of volatile material and the concentration of solid material. Furthermore, undesirable salt effects may be found in soils amended with biochars with high EC values [13].

As expected, the changes in elemental composition reflected differences in the functional groups as identified by solid state ^13^C NMR spectroscopy. OMW was characterized by O-alkyl C (61–90 ppm) and di-O-alkyl-C (91–110) regions, found to be abundant in polysaccharides such as cellulose and hemicellulose [31]. At 300 °C, the signals associated with sugars decreased, but the intensity of the alkyl-C region (0–45 ppm) augmented, a characteristic that is often associated to plant aliphatic compounds (i.e. cutin or wax-rich residues). However, dramatic changes in OMW composition were observed when PT reached 500 °C, 800 °C or 1000 °C. In fact, in correspondence to the severe decrease in N content and H/C ratio, the H- and C-substituted aromatic C fraction (111–140 ppm), as well as the O-substituted aromatic C region (141–160 ppm), increased remarkably. Similarly, we found that many triterpene acids and phenolic compounds disappeared as temperature increased at 300 °C, in favor of the accumulation of fatty acids and dicarboxylic derivatives. These findings have been reported also for other plant residues [45], or when using other analytical approaches [14, 39]. Almendros et al. [38] suggested that the formation of aromatic and heterocyclic N-containing structures may be due to the heating process, whereby the labile compounds are converted into less decomposable ones. However, the alkyl C fraction reduced consistently with pyrolysis at 500 °C. This supports the hypothesis that the relative enrichment of aromatic C compounds reflects both the removal of existing metabolites, but also the production of novel structures, possibly involving previously dehydrated carbohydrates [46]. At temperatures around 300 °C thermal distillation may determine the accumulation of lipids and other aliphatic compounds [46], while subsequent polymerization and transformation of hydrophobic structures may be observed at higher temperatures [47].

LC-MS has been previously applied to characterize bio-oils subjected to pyrolysis [48], a technique that overcomes the limitation of GC-MS, restricted to the analysis only of volatile or semi-volatile compounds [49]. Our data showed that the compounds present in the organic feedstock included phenolic compounds, fatty acids and carboxylic or terpene acids derivatives. The range of molecular masses (186–540) identified by ESI-TOF mass spectrometry agreed with other spectroscopic features of the samples. The detected molecules include carbonyl groups, aromatic rings and aliphatic chains, that may well explain the results obtained by using solid-state ^13^C NMR. It is interesting to note that the majority of the putatively identified molecules were detected in OMW (22 out of 29), while the other compounds were found only in pyrolyzed material at 300 °C, or were in common between these two samples. Thermal treatment at 300 °C reduced the abundance of several compounds, with phenolic compounds totally disappeared while other carboxylic acids derivatives increased. However, the pyrolysis at 500 °C or greater, completely annulled these metabolites from LC-MS chromatograms, thus confirming that high processing temperatures heavily modified biochar composition.

Scanning electron micrographs of the biochars produced at different temperatures also confirmed that the organic samples obtained at 500 °C or greater, were morphologically diverse from both the OMW and biochar produced at 300 °C. SEM analyses revealed changes in the surface and structure of the biochars, with external porosity increasing with temperature. Bonanomi et al. [45] reported that biochar surface morphology was more affected by plant origin than by the processing temperature, therefore the structural change may be related to the dominance of the alkyl rather than the aromatic C groups. In our work, we observed a similar convergence of the chemical composition in pyrolyzed materials, in accordance to previous reports regarding the decomposition of plant litter in natural environments [50, 51].

In multiple bioassays, we tested aqueous extracts from both organic feedstock and burned material at different PTs, assuming that differences among the biochar types corresponded to similar differences among the related extracts. A similar approach was used in previous works [45, 52, 53], and several authors reported beneficial effects of biochar water extracts in terms of improvement of seed germination, plant development and yield, or soil mineral solubilization [54, 55, 56, 57], comparable to those observed with biochars [21, 22]. In this work, the OMW water extracts showed severe inhibition of root growth of both *L*. *sativum* and *B*. *rapa*, in agreement with previous findings regarding crop residues and plant litter [22, 58, 59, 60, 61, 62]. The inhibitory effect was also observed with biochar water extracts produced at 300 °C, but the negative impact drastically reduced in biochar after pyrolysis at temperatures higher than 500 °C. This trend contradicts the hypothesis of nutrient immobilization proposed by [63], that related limited root development to the reduction of available N. In comparison, our results showed that the organic samples containing higher C/N ratios were those associated with biochars produced at PT 500 °C or above, whose water-soluble compounds had a positive effect on plant development. This indicates that mineral N starvation probably is not the cause of root growth inhibition for the selected plants, organic material and bioassay conditions tested. In this regards, C/N ratio does not appear a useful parameter to predict N dynamics in soil and the impact on plant growth. The poor capability of C/N ratio to predict N mineralization or immobilization during organic matter decomposition has been demonstrated for plant litter [64, 65] and biochar [66]. C/N ratio, in fact, has an intrinsic limit because, by taking into account only the total content of organic C, it does not consider its biochemical quality that may be highly variable, ranging from simple sugars, to aromatic and recalcitrant materials. Another factor that influence the inhibitory effect of OMW on root growth may be associated to the release of allelopathic compounds [67, 68]. Interestingly, several studies reported that allelopathic inhibitory effects of plant residues are due to low molecular weight compounds with aromatic C fractions [68, 69]. In contrast, we found negative correlations between alkyl regions of ^13^C NMR spectra and plant growth, whereby aromatic C fractions that were abundant in pyrolyzed biochars were found to be related to plant root development. This apparent contradiction may be explained with the recognised ability of biochar to absorb phytotoxic molecules, thus its presence in the soil-plant zone does not affect mineral nutrition [70, 71]. In fact, due to its sorbent properties, biochar has been also proposed for decontamination of polluted soils and wastewaters [72, 73, 74]. Furthermore, the application of biochar from OMW to contaminated soils was found to reduce mobility, bioavailability and toxicity of metals in agricultural soil, thus improving the quality of the environment [8]. The results found in our present study are consistent with the positive effects of biochar material to work as a soil amendment to improve plant growth and a stabilizer of agro-ecosystems [21], although further experiments are necessary to confirm our results at field scale.

In contrast to higher plants, the effect of the OMW water extracts on the growth of fungi was greater or comparable to those observed in the control conditions. Significant growth inhibition of *Aspergillus*, *Fusarium* and *Trichoderma* was observed with water extracts from all tested pyrolyzed materials that may be attributed to changes in the chemical composition of the organic material, as demonstrated by ^13^C NMR profiles. This effect was also noted for material obtained from burned peat and grass residues [38, 75], where the temperature-dependent reduction of carbohydrates (corresponding to the spectral regions of di-O-alkyl C and O-alkyl C) and the relative increase of aromatic compounds support the hypothesis that microbial growth inhibition may vary according to the availability of easily degradable C sources. Moreover, as previously reported [22] and confirmed in the present study, the addition of an external source of organic carbon and nutrients (PDB) to the biochar water extract treatments was able to restore microbial growth to levels equivalent to that observed in the control (Fig 5B). Therefore, the availability of C sources in undecomposed and thermally treated feedstocks or biochars can directly influence fungal growth. Similarly, Ameloot et al. [76] reported that both the biochar source and composition, as well as the pyrolysis conditions, greatly influenced the properties of the charred material and its effect on soil microbial community. However, other authors [77] stated that the biomass source rather than PT heavily influenced the characteristics of biochars and their agronomic value.

Changes in the suitability of biochar as a substrate for microbial growth could be also related to the presence of fungitoxic compounds that could possibly have an aromatic organic structure as noted in the ^13^C NMR spectra. Finally, the inhibitory effect of the water extracts from the various organic materials may also depend upon the specific response of given fungi or the diverse combinations of the feedstocks and biochars to the fungal species. For example, present findings indicated no inhibition of mycelium growth for *R*. *solani* with either OMW or biochars water, even though previous studies reported inhibition of both *Pythium* and *Rhizoctonia* growth with lignified, tannin rich litters [78, 79]. Correlations of the chemical parameters of the OMW and the biochars from varying PTs to the performances of organisms belonging to the different biological groups suggest opposing growth activity responses of the fungi as compared to the plants or the nematode *M*. *incognita* (Fig 7). A difference was noted for the soilborne pathogen *R*. *solani*, which showed a potentially closer correspondence to the growth preferences observed for the nematode or the plants. Nevertheless, the correlation analysis aims only to infer about possible association between bioassay results and the chemical quality of the organic materials. Indeed, further data based on a larger sample size would be necessary to validate such relationships.

The overall effect of *in vitro* applications of water extracts from the organic materials to *M*. *incognita* was similar to that observed for plants, whereby extracts from untreated OMW or biochar at PT 300 °C produced total inhibition of the nematode after 10 days, and impeded plant root growth. In the chemical analysis of OMW and biochar at PT 300 °C, several compounds were detected, mainly fatty acids and phenols, that are known to be among the phytochemical compounds that demonstrate nematicidal effects [80]. The presence of these metabolites was found to reduce in the biochars subjected to increasing PTs and, correspondingly, diminished the inhibition on nematode mobility. Nico et al. [81] suggested that mature compost is less effective in controlling root-knot nematodes because the decomposition process reduces the presence of compounds with inhibitory effects over time. In our study, *M*. *incognita* response varied significantly according to the composition of the tested extracts, suggesting that the observed nematicidal effect may be related to the presence of specific compounds released by OMW or biochars. These evidences are in accordance with other studies reporting that disease control in amended soil is the result of the ability of crop residues or biochars to release toxic compounds (i.e. pesticides, metals, etc.) that affect soil microfauna and microflora [82, 83]. However, to clarify the effect of biochars on *M*. *incognita* or other phtyoparasitic nematode species further studies are necessary.

## Conclusions

This study provides evidence that pyrolysis induced radical changes in the chemical composition of solid OMW, and that the properties of the resulting biochars were influenced by thermal process. The increase of PT determined a progressive loss of alkyl C fractions and a corresponding increase of aromatic ones in the corresponding biochar. Similarly, biochars obtained at PT ≥ 500 °C showed similar structural aggregations and a consistent reduction of C = O-containing compounds. In multiple bioassays, water extracts of untreated OMW had an inhibitory effect on selected plant species as well as on the nematode *M*. *incognita*, while stimulated fungal growth. Conversely, after pyrolysis, we observed a reduction of degradable carbon sources and an increase of aromatic fractions that can be possibly associated to plant and nematode growth and to inhibition of most of the tested fungal species. Our results, even being limited by the use of water extracts and a restricted range of species studied, suggest that the pyrolysis process may be used to transform OMW into valuable products that can be successfully applied as soil amendments. However, further laboratory, greenhouse and field experiments are indeed necessary to validate this hypothesis.

In conclusion, this investigation illustrates the importance of studying how the manipulation of recovered organic wastes, recycled in support of the circular economy, can influence the properties of the end-products. The use of multiple analytical approaches to characterize organic wastes may be of fundamental importance for the consideration of their potential successful applications in the development of a sustainable agricultural system.
